# NEDD4-mediated HSF1 degradation underlies α-synucleinopathy

**DOI:** 10.1093/hmg/ddv445

**Published:** 2015-10-26

**Authors:** Eunhee Kim, Bin Wang, Namratha Sastry, Eliezer Masliah, Peter T. Nelson, Huaibin Cai, Francesca-Fang Liao

**Affiliations:** 1 Department of Pharmacology and; 2 Department of Anatomy and Neurobiology, University of Tennessee Health Science Center, 874 Union Avenue/Crowe 401, Memphis, TN 38163, USA,; 3 Transgenics Section, National Institute on Aging, National Institutes of Health, Bethesda, MD 20892, USA,; 4 Department of Neurology, Sanders-Brown Center on Aging, 800 South Limestone Street, Lexington, KY 40536, USA and; 5 Department of Neurosciences, University of California San Diego, La Jolla, CA 92093, USA

## Abstract

Cellular protein homeostasis is achieved by a delicate network of molecular chaperones and various proteolytic processes such as ubiquitin–proteasome system (UPS) to avoid a build-up of misfolded protein aggregates. The latter is a common denominator of neurodegeneration. Neurons are found to be particularly vulnerable to toxic stress from aggregation-prone proteins such as α-synuclein. Induction of heat-shock proteins (HSPs), such as through activated heat shock transcription factor 1 (HSF1) via Hsp90 inhibition, is being investigated as a therapeutic option for proteinopathic diseases. HSF1 is a master stress-protective transcription factor which activates genes encoding protein chaperones (e.g. iHsp70) and anti-apoptotic proteins. However, whether and how HSF1 is dysregulated during neurodegeneration has not been studied. Here, we discover aberrant HSF1 degradation by aggregated α-synuclein (or α-synuclein-induced proteotoxic stress) in transfected neuroblastoma cells. HSF1 dysregulation via α-synuclein was confirmed by *in vivo* assessment of mouse and *in situ* studies of human specimens with α-synucleinopathy. We demonstrate that elevated NEDD4 is implicated as the responsible ubiquitin E3 ligase for HSF1 degradation through UPS. Furthermore, pharmacologically induced SIRT1-mediated deacetylation can attenuate aberrant NEDD4-mediated HSF1 degradation. Indeed, we define the acetylation status of the Lys 80 residue located in the DNA-binding domain of HSF1 as a critical factor in modulating HSF1 protein stability in addition to its previously identified role in the transcriptional activity. Together with the finding that preserving HSF1 can alleviate α-synuclein toxicity, this study strongly suggests that aberrant HSF1 degradation is a key neurodegenerative mechanism underlying α-synucleinopathy.

## Introduction

Synucleinopathies are a major class of neurodegenerative diseases, including Parkinson's disease (PD) and diffuse lewy body (DLB). These pathologic conditions are defined by the presence of pathological α-synuclein aggregates known as Lewy bodies. Disruption of protein quality control, interconnected cellular strategies of the ubiquitin–proteasome system (UPS) and molecular chaperones, has been postulated to be involved in the pathogenesis of neurodegeneration underlying proteinopathies (1). Molecular chaperones composed of a set of heat shock proteins (HSPs) are considered a first line of defense against misfolded and aberrantly accumulated proteins like α-synuclein aggregates that trigger the pathologic cascade and eventually lead to deficits in the overall protein clearance. For unknown mechanism, several cell types including neurons appear to be poorly adapted for chronic proteotoxic stress (2).

Since the induction of HSPs is primarily determined by the activation of heat shock transcription factor 1 (HSF1), HSF1 has been regarded as an attractive target for treating neurodegenerative diseases (3). Multiple post-translational modifications of HSF1 have been studied in relation to its activity state, including acetylation, sumoylation and phosphorylation (4–8). However, regulation of HSF1 turnover remains relatively unexplored, especially in neurons. It should be noted that there have been increasing reports on linking the intracellular level of HSF1 with neuronal survival/death (9–12), suggesting that HSF1 loss can be a major contributor to neurodegeneration.

In this work, we discovered the ‘neural precursor cell expressed, developmentally down-regulated 4 (NEDD4-1, NEDD4 herein)’ (13,14) likely being the E3 ligase of HSF1 in neurons under proteotoxic stress conditions. We speculate that aberrant HSF1 degradation is a common and important key molecular mechanism underlying neurodegeneration. Moreover, HSF1 deacetylation can attenuate this process via stabilizing HSF1 protein.

## Results

### Overexpressed α-syn protein promotes ubiquitination and degradation of HSF1 protein via UPS

**Figure 1.**
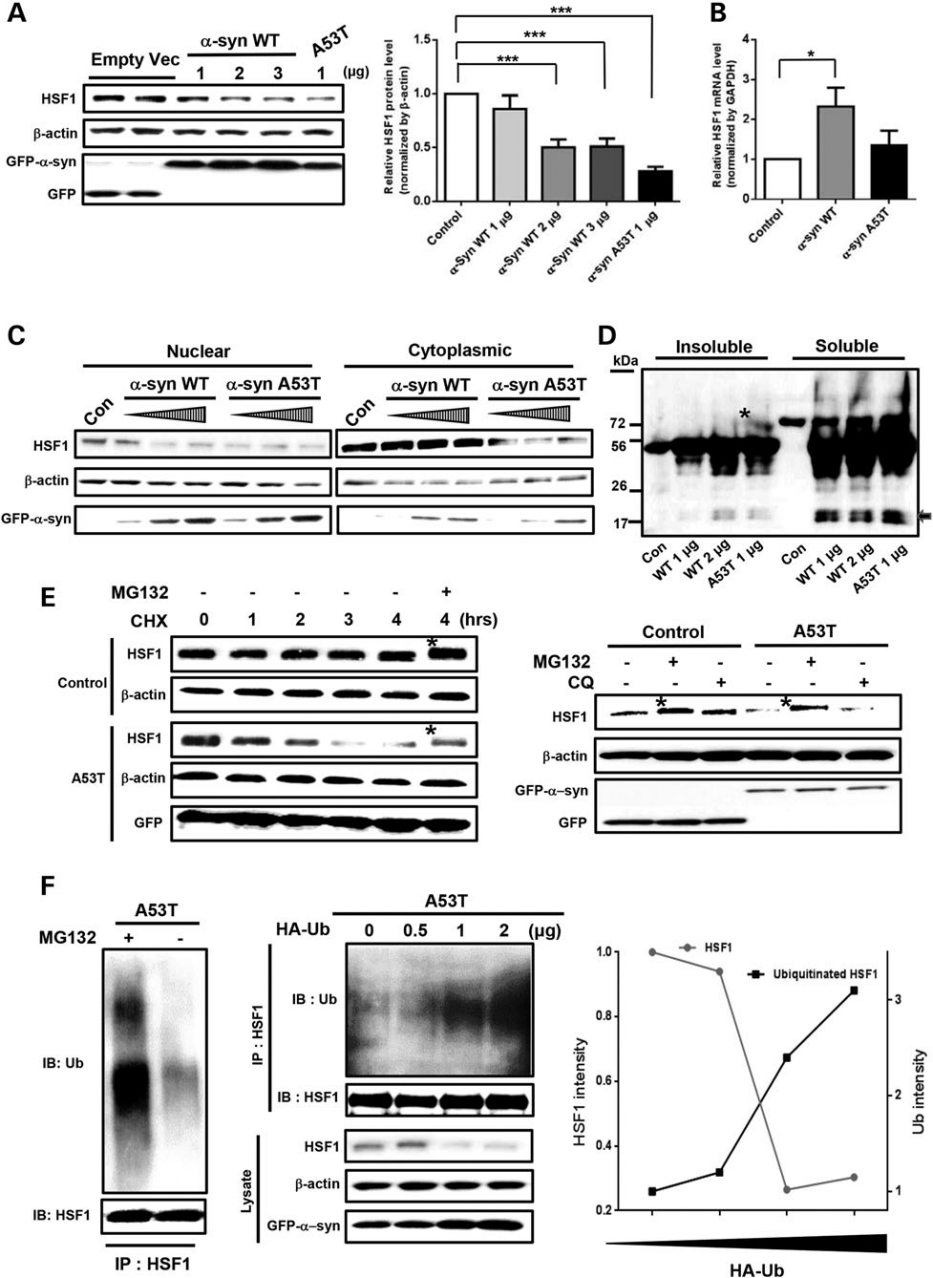
α-Syn aggregation causes HSF1 degradation via UPS. (**A**) Immunoblot assay showing the expression levels of HSF1. SH-SY5Y cells were transiently transfected with different concentrations of WT α-syn or A53T α-syn plasmids. Quantification of the western blot images was done by densitometry (right, *n* = 3, ***P < 0.001, means ± SD). (**B**) Quantitative measurement of HSF1 mRNA levels normalized by GAPDH transcripts in transfected SH-SY5Y cells by qRT-PCR (*n* = 3, ***P < 0.001; *P < 0.05, data represent means ± SD). (**C**) Subcellular fractionation assay to separate nuclear and cytoplasmic HSF1. Cells were transfected with different amounts of α-syn (WT or A53T) constructs. (**D**) Following Triton X-100 fractionation, both insoluble and soluble α-syn were detected by using α-syn antibody instead of GFP antibody in transfected SH-SY5Y cells (arrow represents monomeric α-syn of 18 kDa size. *tetramer of α-syn). (**E**) Proteosomal HSF1 degradation induced by A53T α-syn. SH-SY5Y cells were treated with 50 μM CHX for various time points with or without pretreatment of 25 μM MG132, 48 h after transfection. MG132 but not CQ (50 μM) blocked HSF1 degradation (right panel). Control was transfected with empty vector (A–E). (**F**) HSF1 ubiquitination in A53T α-syn transfected cells. Co-immunoprecipitation of ubiquitin (Ub) with HSF1 using antibody against HSF1 was performed with protein extracts of A53T-transfected SH-SY5Y cells with or without MG132 treatment (left panel). GFP-α-syn A53T was transiently co-transfected with various amounts of HA-tagged ubiquitin (HA-Ub) into SH-SY5Y cells (right panel). The HSF1-complexes immunoprecipitated from lysates with anti-HSF1 antibody were then subjected to immunoblot using an antibody against ubiquitin. HSF1 abundance showed an inverse relationship to the level of ubiquitination.

**Figure 2.**
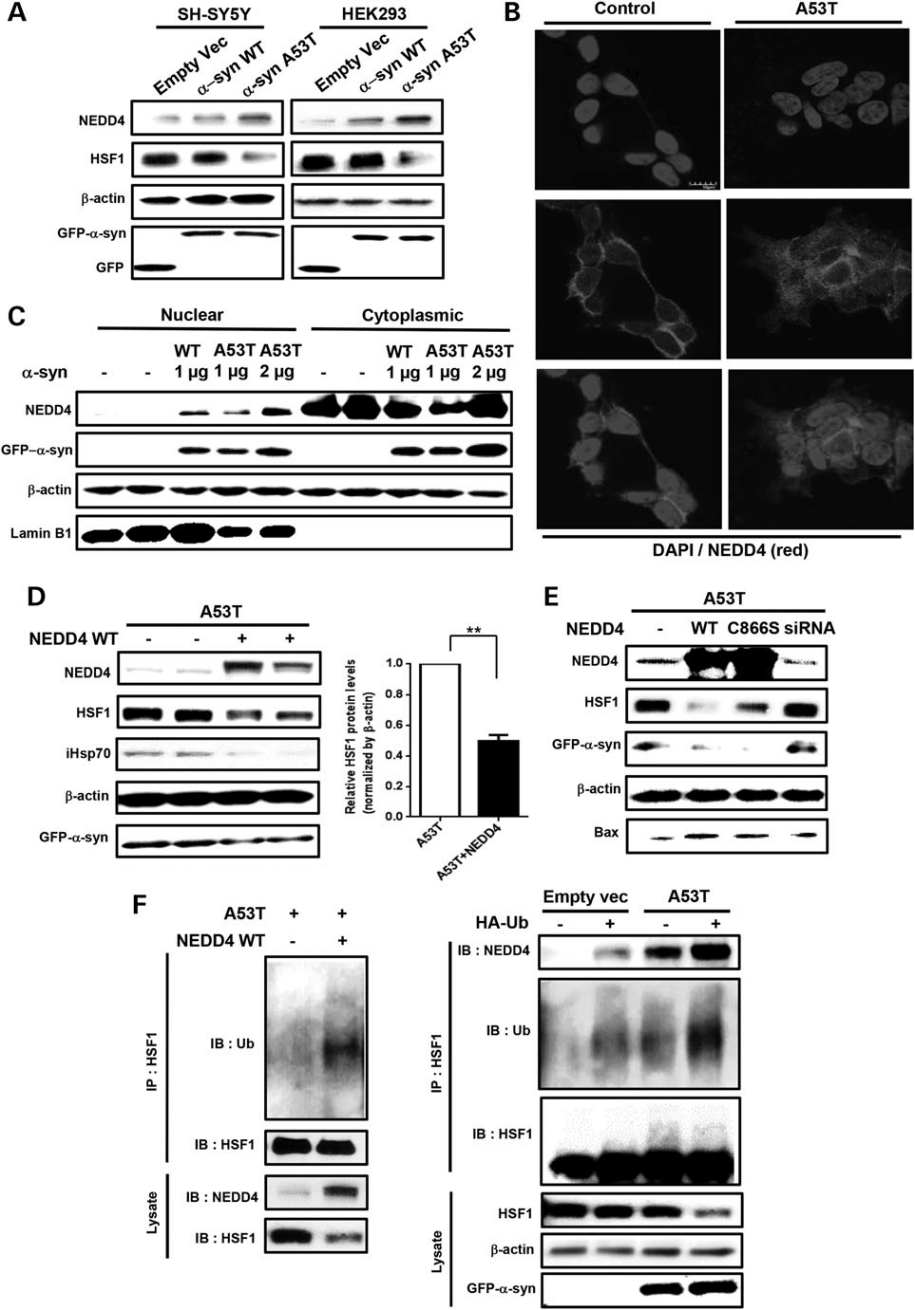
NEDD4 promotes ubiquitination and proteasomal degradation of HSF1 in neuroblastoma cells overexpressing A53T α-syn. (**A**) Elevated NEDD4 and decreased HSF1 protein levels by A53T α-syn overexpression in SH-SY5Y and HEK293 cells. Cells were transfected with empty GFP vector (control), α-syn WT or α-syn A53T. (**B**) Intracellular NEDD4 protein expression (red) in SH-SY5Y cells with or without A53T α-syn was visualized by immunocytochemistry. DAPI (blue) was used for nuclear staining. The merged images in the bottom. Scale bar: 10 μm. (**C**) Subcellular fractionation assay to separate nuclear and cytoplasmic NEDD4. SH-SY5Y cells were transfected with α-syn (WT or A53T). Lamin B1, a nuclear envelope marker, was shown to indicate nuclear fraction. (**D** and **E**) NEDD4 WT overexpression in SH-SY5Y cells transfected with A53T α-syn further decreases HSF1, inducible Hsp70 and α-syn levels and increases expression of pro-apoptotic factor Bax. SH-SY5Y cells were transfected with A53T α-syn, along with NEDD4 WT, NEDD4 C866S or NEDD4 siRNA. Quantification of HSF1 protein expression was normalized to β-actin (*n* = 3, **P < 0.01, means ± SD). (**F**) NEDD4 WT overexpression facilitates ubiquitination of HSF1 in A53T-transfected cells (left panel). The interaction between NEDD4 and HSF1 (right panel). Three days after transfection of GFP-A53T or empty GFP vector alone or together with HA-Ub in SH-SY5Y cells, endogenously expressed HSF1 proteins were pull-down in whole-cell lysates, followed by immunoblot using anti-Ub and anti-NEDD4 antibody (right panel).

To determine whether α-synuclein (α-syn, herein after) aggregation altered HSF1 expression levels, we transiently transfected SH-SY5Y neuroblastoma and HEK293 cells with GFP-tagged wild-type (WT) α-syn (GFP-α-syn WT) or GFP-tagged A53T mutant α-syn (GFP-α-syn A53T). In both cell lines, A53T α-syn overexpression 48 h post transfection caused more dramatic reduction (>70% loss) in HSF1 protein expression than WT α-syn (Figs 1A, C and 2A), without decreasing the mRNA levels of *hsf1* gene (Fig. 1B). Similar results were obtained when HSF1 protein levels were determined at 24 or 72 h after transfection (Supplementary Material, Fig. S1). A53T mutant form of α-syn is known to aggregate more rapidly than WT form. We could detect both Triton X-soluble and detergent-insoluble α-syn aggregates in α-syn transfected SH-SY5Y cells (Fig. 1D). Insoluble WT α-syn expression was increased by transfection of double concentration of WT α-syn (WT 2 μg) (Fig. 1D). This higher WT α-syn aggregation resulted in ∼50% loss of HSF1 protein (Fig. 1A and C). In contrast to A53T α-syn which resulted in HSF1 loss in both nuclear and cytoplasmic compartments, WT α-syn only caused reduced HSF1 in the nucleus (Fig. 1C). HSF1 loss appears to be a common phenomenon upon proteotoxic stress since other aggregation-prone proteins such as mutant huntingtin (Htt), but not transactive response DNA-binding protein (TDP-43), also led to reduced HSF1 protein levels (Supplementary Material, Fig. S2).

We next assessed HSF1 protein stability by treating transfected cells with cyclohexamide (CHX). While HSF1 protein was stable in SH-SY5Y cells transfected with empty vector, it was greatly reduced in cells overexpressing A53T over time (Fig. 1E), which was largely rescued by proteasome inhibitor MG132 but not by chloroquine (CQ, a lysosomal inhibitor). Of note, we noticed upshifted HSF1 bands in both control and A53T-trasnfected cells when blocking proteasomal degradation with MG132 (Fig. 1E; denoted by *). This slower mobility-shifted band may be due to hyperphosphorylated HSF1 which correlated with hyperactivated HSF1 following heat shock and proteasomal stress (15).

Polyubiquitination is a pre-requisite step for proteasome system to degrade target proteins. We found that HSF1 proteins to be degraded by proteasome were indeed highly polyubiquitinated, as revealed by MG132 treatment (Fig. 1F). HSF1 ubiquitination was further proven to be involved in HSF1 degradation by *in vivo* ubiquitination assay. As HA-Ub was increasingly acquired in A53T-transfected cells, HSF1 protein became more polyubiquitinated, correlating with decreased HSF1 protein levels (Fig. 1F). On the contrary, GFP-α-syn A53T expression was increased by exogenous ubiquitin overexpression, consistent with a positive role of HSF1 in α-syn clearance (Fig. 6).

### α-Syn-induced HSF1 degradation is mediated by the E3 ligase NEDD4

UPS degradation is mediated by ubiquitin E3 ligase conjugating ubiquitin molecules to a lysine residue of substrate protein. Previously, we discovered that E3 ubiquitin-protein ligase NEDD4 expression was upregulated in the brain samples of PD patients (16). The LPKY-motif in the endoplasmic reticulum localized transcription factor Spt23p was previously shown to interact with yeast Rsp5 (mammalian homolog NEDD4) (17). We found the same motif in the DNA-binding domain of HSF1 (57–60 amino acid sequence). Therefore, we reasoned whether NEDD4 may be involved in HSF1 protein degradation. Indeed, we observed significant enhancement of NEDD4 expression and in particular nuclear NEDD4 during α-syn-induced HSF1 degradation (Fig. 2A–C). NEDD4 appeared to undergo active shuttling between nuclear and cytoplasmic compartments upon overexpressing A53T as compared with its perinuclear distribution in control cells, as detected by both subcellular fractionation assay and confocal microscopy (Fig. 2B and C).

To investigate NEDD4-mediated HSF1 degradation, we overexpressed WT NEDD4 (NEDD4 WT) in A53T-transfected SH-SY5Ycells. It should be noted that A53T-transfected SH-SY5Ycells overexpressing NEDD4 WT showed ∼50% reduction of HSF1 along with decreased inducible Hsp70 (iHsp70) whose expression is regulated by HSF1 activation (Fig. 2D). Interestingly, we could not see any effect of NEDD4 overexpression on HSF1 expression levels in control cells (Supplementary Material, Fig. S3). On the contrary, overexpression of catalytically defective NEDD4 mutant (NEDD4 C866S) as well as NEDD4 siRNA failed to reduce HSF1 protein (Fig. 2E), indicating that the ubiquitin ligase activity of NEDD4 was required for the loss of HSF1 protein. We then detected enhanced mono-and polyubiquitination of HSF1 in SH-SY5Y cells overexpressing NEDD4 WT (Fig. 2F) which was further facilitated by expression of exogenous HA-Ub in both control and A53T-transfected cells (Fig. 2F). Moreover, the strength of NEDD4–HSF1 interaction appeared to be positively correlated with the degree of HSF1 polyubiquitination and its degradation (Fig. 2F).

Taken together, we could conclude that despite the reduction in α-syn (Figs 2D, E and 6A, B), degradation of HSF1 was further promoted by NEDD4 overexpression in A53T-transfected SH-SY5Y cells.

### Loss of HSF1 protein induced by α-synucleinopathy *in vivo*

**Figure 3.**
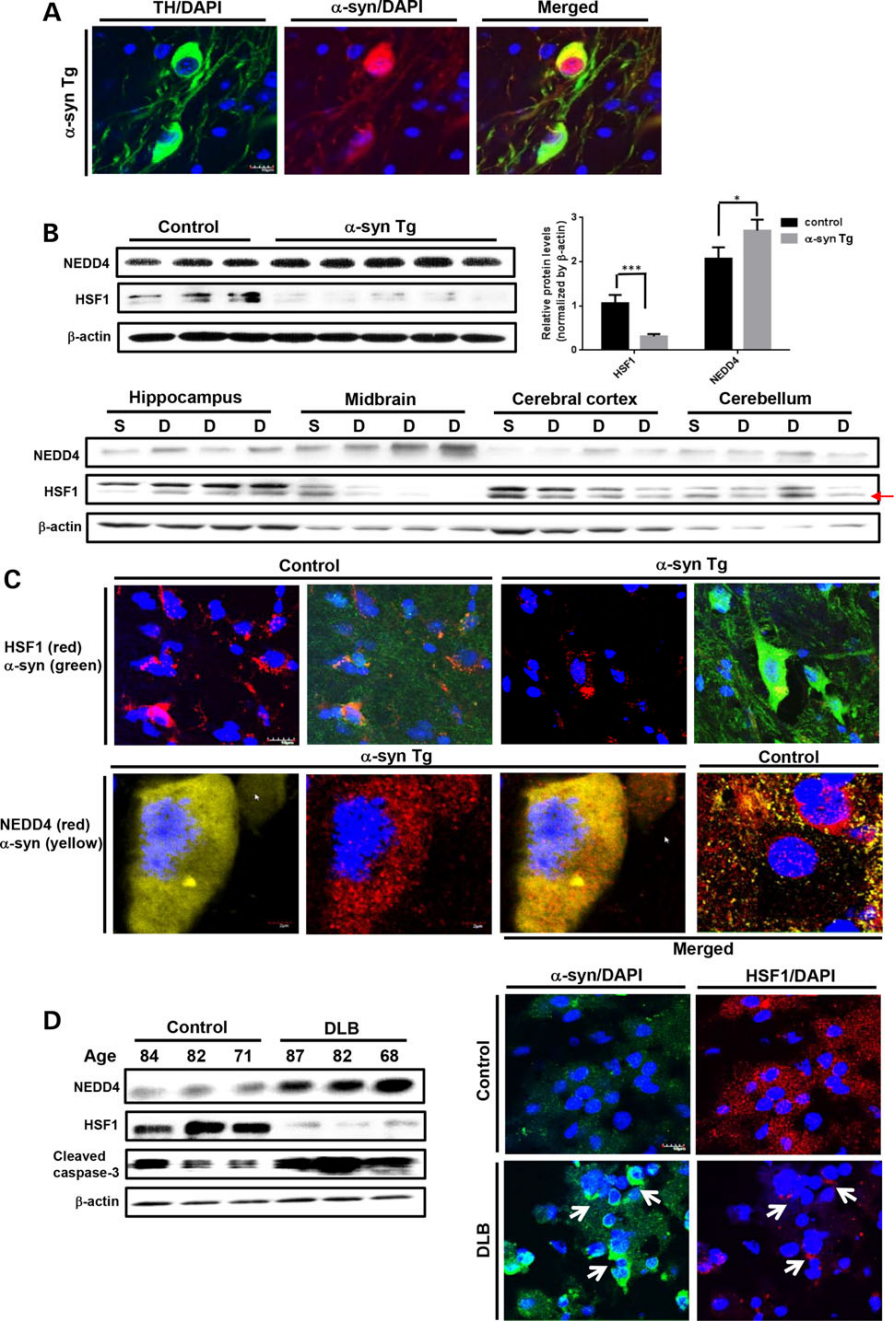
Reciprocal NEDD4 and HSF1 levels in mouse and human tissue of α-synucleinopathy. (**A**) Somatic α-syn overexpression (red) in the substantia nigra of α-syn double transgenic mice. Green and yellow indicate TH-positive cells and those with somatic α-syn aggregation, respectively. Scale bar: 10 μm. (**B**) (Top panels) Western blot analysis of the midbrain tissue of TetO-α-syn single and α-syn double transgenic mice (12 months of age). Quantification of HSF1/NEDD4 protein expression (***P < 0.001, *P < 0.05, means ± SD). (Bottom panels) Western blots on four different areas of the brain (i.e. hippocampus, midbrain, cerebral cortex and cerebellum) with equal loading of total proteins. S, single transgenic mice (control); D, double transgenics (α-syn Tg). The red arrow indicates the predicted molecular weight of HSF1 (82 kDa). (**C**) Confocal microscopy of the substantia nigra from single and double α-syn transgenic mice. Top panels: HSF1 (red); α-syn (green). Bottom panels: NEDD4 (red); α-syn (yellow). Scale bar: 10 μm (top panels) and 2 μm (bottom panels). (**D**) Western blot analysis of the inferior parietal lobes of human patient specimens with and without diffuse Lewy body disease (left panel). Immunohistochemistry of HSF1 (red) and α-syn (green) (right panel). Scale bar: 10 μm.

**Figure 4.**
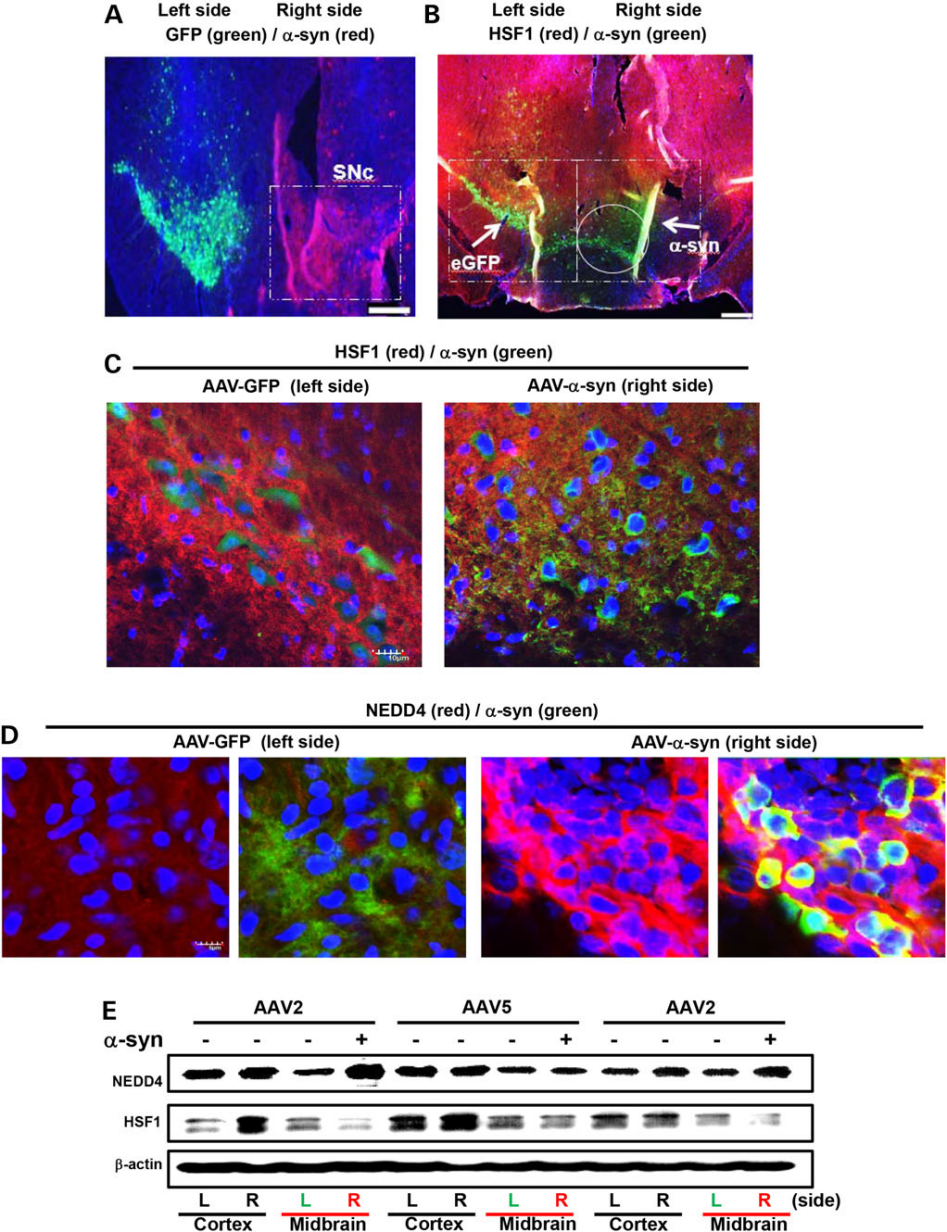
HSF1 degradation induced by AAV-α-syn viruses in mouse substantia nigra. Young adult C57BL/6 mice were injected with AAV-α-syn or control eGFP viruses each side of SNc (*n* = 4). (A–D) HSF1, NEDD4 and α-syn levels were detected by immunohistochemistry. (**A**) Representative image of both sides of substantia nigra (SNc) is shown, each receiving AAV2-eGFP control (left side) or AAV2-α-syn viruses (right side). White dotted boxes indicate the SNc regions. Scale bar: 500 μm. (**B**) Coronal brain slices were stained for HSF1 (red) and α-syn (green); immunopositive signals are indicated by white arrows within the SNc regions. White circle shows that the α-syn-positive region is devoid of red HSF1 signal. Scale bar: 1 mm. (**C** and **D**) The same brain slice from panel B was examined under confocal microscope, showing immunopositive signals of HSF1, NEDD4 and α-syn with higher magnification. Scale bars: 10 μm (C) and 5 μm (D). (**E**) Western blot analysis of the cortex (unaffected area used as control) and midbrain where SNc is located of two AAV2-α-syn injected mice and one AAV5-α-syn injected mouse. L, left side injected with AAV-GFP virus; R, right side subjected to AAV-α-syn virus injection.

We determined the protein expression profiles of HSF1 and NEDD4 in the brain of *PITX3-IRES2-tTA/tetO-a-syn* inducible double transgenic mouse expressing WT α-syn under the PITX3 promoter predominantly expressed in midbrain dopaminergic neurons. *TetO-α-syn* single transgenic mouse was used as a control to compare with double transgenic mice overexpressing α-syn mostly in the midbrain area. By immunostaining, we confirmed somatic accumulation of α-syn in tyrosine hydroxylase (TH)-positive dopaminergic neurons in midbrain substantia nigra of 12-month-old double transgenic mouse (Fig. 3A). This abnormal accumulation of α-syn in cell bodies was recently identified to be correlated with progression of neurodegeneration in α-syn transgenic mice (18). Our results showed that the most reduction in HSF1 protein was detected in the midbrain samples of double transgenic mice where α-syn was highly overexpressed, accompanied by strongly elevated NEDD4 in those compared with control (Fig. 3B and C). However, the other three regions, hippocampus, cortex and cerebellum, did not show significant changes (Fig. 3B). Interestingly, we observed two bands of HSF1 proteins only in mouse tissues; the upper band is higher than its predicted molecular weight of 82 kDa which might be due to by post-translational events such as phosphorylation (Figs. 3B and 4E). Although NEDD4 expressed primarily in the perinucleus of neurons in the age-matched control mice, we observed that NEDD4 was enormously induced in the soma of cells where α-syn accumulated in the brain slices of α-syn transgenic mice by immunohistochemistry (Fig. 3C).

While Lewy body in PD is primarily found in the midbrain substantia nigra and locus ceruleus, DLB is also categorized as α-synucleinopathy present in the subcortical and cortical (frontotemporal) regions of the brain. We observed a striking decrease in HSF1 and NEDD4 upregulation within the inferior parietal lobes of human patients with DLB where Lewy body deposits were located (Fig. 3D). Cleaved-caspase 3 as a marker of apoptosis was highly expressed in the individuals with reduced HSF1 (Fig. 3D, left panel). Immunostaining of HSF1 showed a punctate pattern which colocalized with aggregated α-syn, likely indicating stress granules (Fig. 3D, right panel).

Since reciprocal expression levels of NEDD4 and HSF1 were found in mouse and human tissue of α-synucleinopathy, we then sought *in vivo* evidence of α-syn-induced HSF1 degradation. Six-month-old C57BL/6 mice were injected with AAV2 or AAV5-CBA-α-syn in the right substantia nigra and AAV-CBA-eGFP virus in the left side as control (Fig. 4A). Remarkably, 3 weeks later, drastic HSF1 degradation was detected only in the right side of substantia nigra overexpressing α-syn (Fig. 4B and C), accompanied by markedly increased NEDD4 which appeared to colocalize with α-syn around perinucleus (Fig. 4D). In addition to immunohistochemistry, 5–6 weeks after injection, the cortex and midbrain samples of two AAV2-CBA-α-syn injected mice and one AAV5-CBA-α-syn injected mouse were subjected to the western blot, which clearly demonstrated HSF1 degradation and NEDD4 elevation in the targeted midbrain area of AAV2-α-syn injected mice (Fig. 4E).

### The role of acetylation status in NEDD4-mediated HSF1 degradation via interplay with ubiquitination

**Figure 5.**
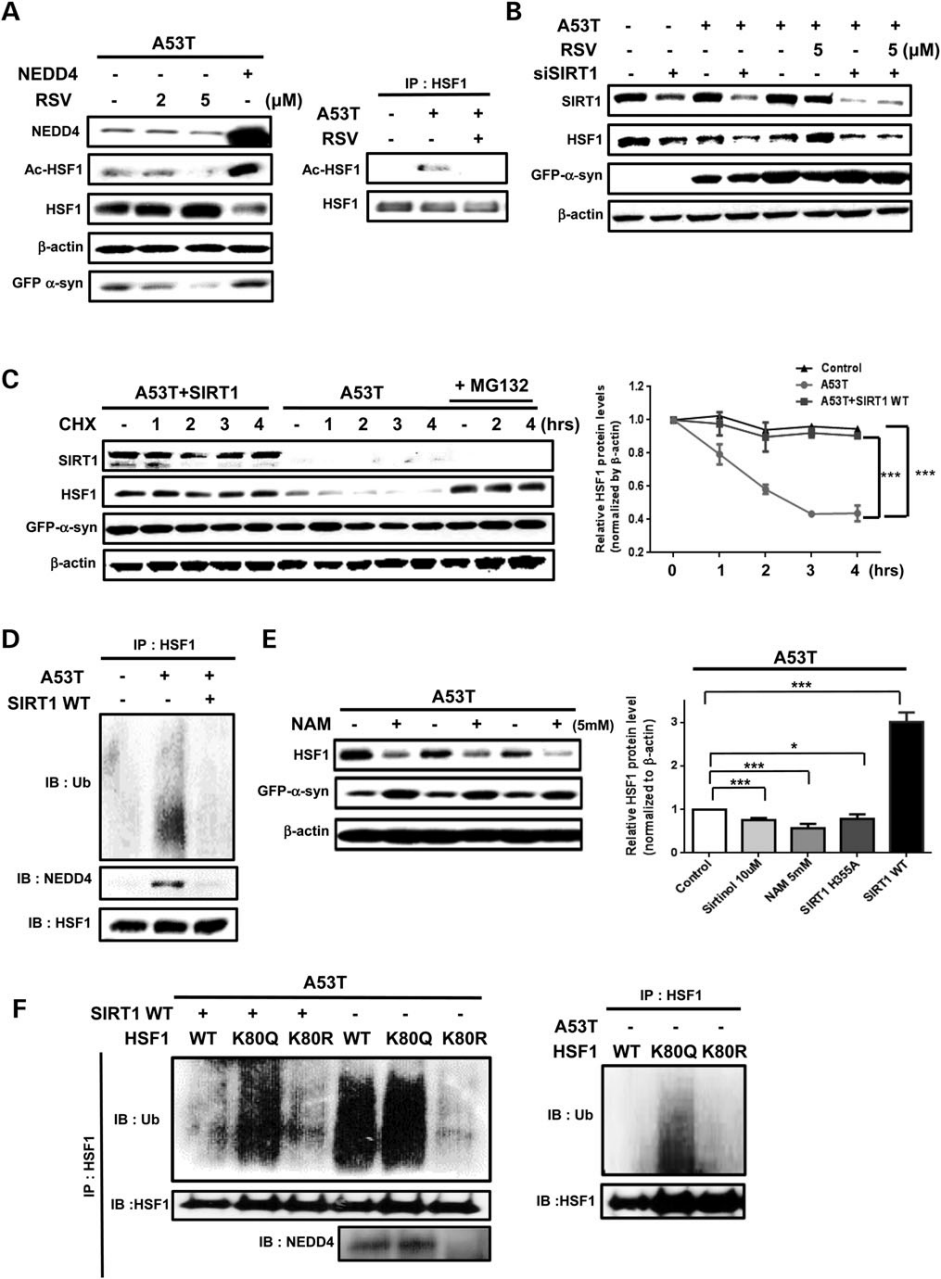
HSF1 acetylation/deacetylation and ubiquitination. (**A** and **B**) SH-SY5Y cells were co-transfected with NEDD4 WT plasmid or SIRT1 siRNA (B) for 48 h with or without co-treatment of RSV. After transfection and RSV treatment, cell lysates were analyzed by acetylation assay using immunoprecipitation and western blot. (**C** and **D**) Effects of SIRT1 WT overexpression on HSF1 deacetylation, ubiquitination and NEDD4 interaction with HSF1. (**E**) Effect of 5 mm NAM on HSF1 and α-syn protein expression levels. Quantification of HSF1 protein levels normalized to β-actin in cells treated with 10 μM sirtinol and 5 mm NAM, and overexpressing SIRT1 H355A (*n* = 3, *P < 0.05, ***P < 0.001, means ± SD). (**F**) Cells overexpressing A53T with or without SIRT1 WT were co-transfected with HSF1 WT or HSF1 K80Q or HSF1 K80R. The resulting cell lysates were subjected to *in vivo* ubiquitination assay. Endogenous HSF1 was pulled down by HSF1 antibody and HSF1-complex was subjected to immunoblot with anti-NEDD4 and anti-ubiquitin antibodies. *In vivo* ubiquitination assay in control cells without A53T mutation (right panel).

In addition to ubiquitination, acetylation of HSF1 was remarkably elevated by A53T α-syn aggregation in SH-SY5Y cells (Fig. 5A). Resveratrol (RSV) has been shown to protect neurons against α-syn toxicity in a SIRT1-dependent mechanism (19). We investigated the effect of RSV on HSF1 levels as well as on its acetylation state. We found that 5 μM of RSV was able to decrease α-syn expression and increase HSF1 protein levels while decreasing HSF1 acetylation levels (Fig. 5A). Most strikingly, NEDD4 overexpression resulted in reduced HSF1, correlating with highly acetylated HSF1. The RSV's effect on reducing HSF1 acetylation appeared to be dependent on its action on SIRT1 activation since co-transfection of A53T α-syn with SIRT1 siRNA completely abolished its beneficial effect (Fig. 5B). The tight regulation of HSF1 expression levels by SIRT1 was further confirmed by adding sirtuin inhibitors, sirtinol or nicotinamide (NAM), or co-transfection with a dominant-negative (DN) SIRT1 H355A defective in catalytic function (Fig. 5E and Supplementary Material, Fig. S4A). Furthermore, SIRT1 WT overexpression prevented HSF1 degradation in A53T-transfected cells without altering HSF1 mRNA levels (Fig. 5C and Supplementary Material, Fig. S4B). SIRT1 overexpression led to reduced interaction between HSF1 and NEDD4 as well as decreased polyubiquitination of HSF1 (Fig. 5D). Taken together, HSF1 acetylation status appeared to be positively correlated with its ubiquitination but negatively with HSF1 protein stability under both basal and A53T-stressed conditions.

K80 is the lysine site located in the DNA-binding domain of HSF1 where its deacetylation was found to increase DNA-binding activity on the promoters of HSP genes (20). Therefore, it has been indirectly implicated as the major target by SIRT1 whose activation causes a delay in the attenuation of heat shock response (HSR). Given the positive role of SIRT1 in stabilizing HSF1 protein, we sought to determine if K80 is also responsible for the underlying mechanism. We replaced K80 with either glutamine (K80Q) to mimic acetylation or arginine (K80R) as non-acetylatable mutation. Results from the CHX experiment indicated that the extent of HSF1 degradation was minimized in A53T-transfected SH-SY5Y cells overexpressing K80R as compared with K80Q (Supplementary Material, Fig. S4C). Accordingly, while HSF1 K80R was completely resistant to ubiquitination, HSF1 K80Q was highly mono- and poly-ubiquitinated than HSF1 WT was (Fig. 5F). This tendency of HSF1 ubiquitination upon K80 residue was preserved in SIRT1-overexpressing A53T-transfected cells (Fig. 5F). Similarly, under basal condition without A53T stress, only K80Q mutation caused HSF1 polyubiquitination (Fig. 5F).

As our *in vitro* study reveals the importance of SIRT1 in HSF1 protein stability, we observed reduced SIRT1 protein levels in the midbrain area of both α-syn transgenic mice and the inferior parietal lobes of human patients with DLB (Supplementary Material, Fig. S5A and B), all of which were previously identified to be lack of HSF1 protein (Figs 3 and 4), although the midbrain of the AAV-α-syn injected mice did not show any change in SIRT1 expression levels (Supplementary Material, Fig. S5C). More strikingly, SIRT1 expression was reduced in the tissues of human DLB patients at a similar level to HSF1 (Supplementary Material, Fig. S5B).

### Downregulation of NEDD4 or overexpression of HSF1 or RSV provides neuroprotection against α-synucleinopathy

**Figure 6.**
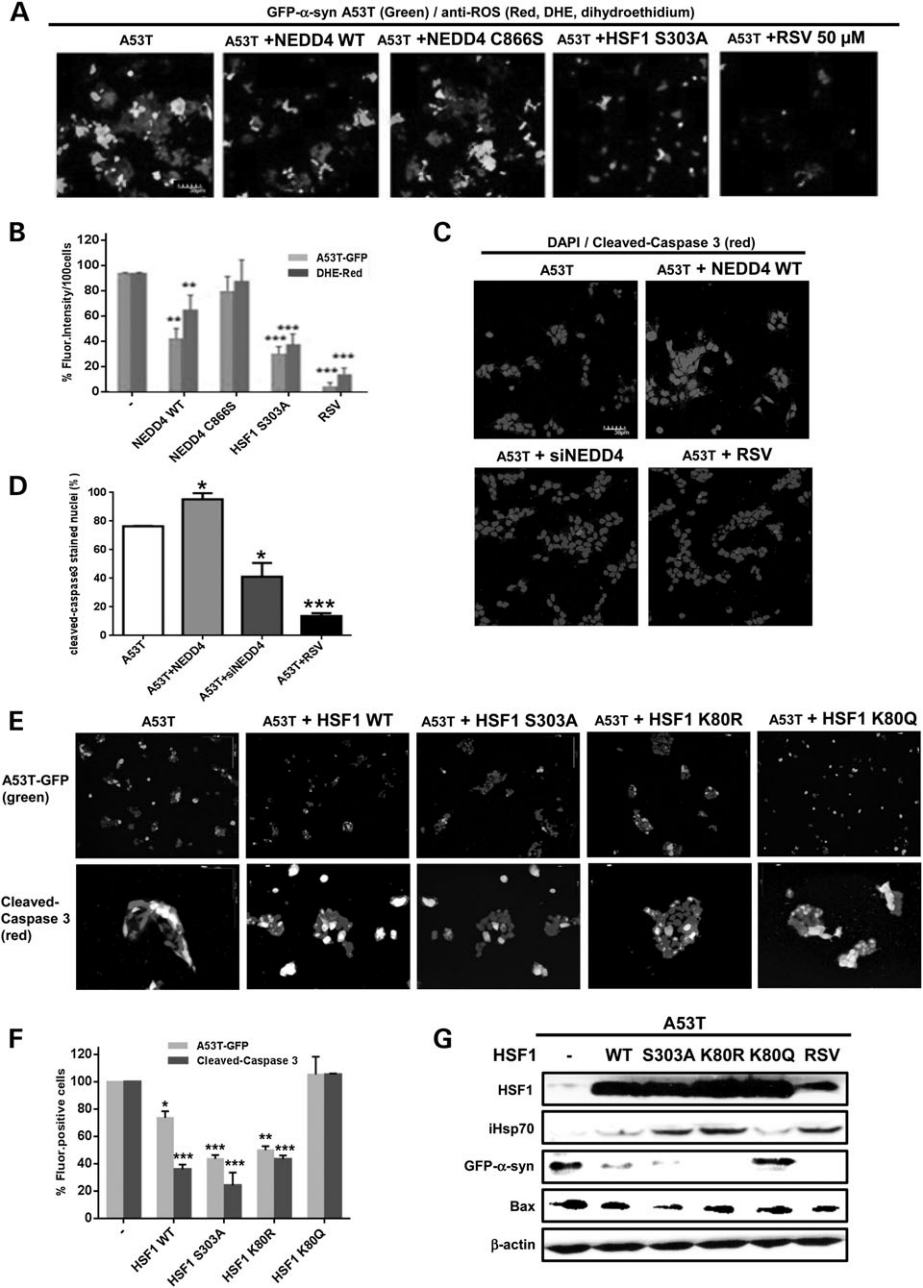
Effects of down-regulated NEDD4 or overexpressed HSF1 or RSV on α-syn toxicity. (**A** and **B**) ROS production was determined by DHE staining (red) for different groups of cells. Green indicates GFP-A53T α-syn expression. A53T α-syn transfected SH-SY5Y cells were co-transfected with NEDD4 WT, NEDD4 C866S or HSF1 S303A (constitutively activated) or treated with RSV 50 μM. Scale bar: 30 μm (A). Graph indicates the percentage of fluorescence intensity per 100 cells (B). (**C** and **D**) Apoptotic cells identified by positive cleaved caspse-3 staining (red) with DAPI nuclear staining (blue). A53T α-syn transfected SH-SY5Y cells were co-transfected with NEDD4 WT or NEDD4 siRNA, or treated with RSV 20 μM. Quantification of cleaved-caspase 3 stained nucleus for each group, represented as percentage on the graph (D). Scale bar: 30 μm (B and D graphs, *n* = 4, *P < 0.05, **P < 0.01, ***P < 0.001, means ± SD). (**E** and **F**) The effect of WT and different mutant forms of HSF1 overexpression on A53T α-syn-induced apoptosis was determined by positive cleaved caspase-3 signals. (E) Representative image of GFP-A53T α-syn (top panels) and cleaved caspase-3 staining (bottom panels) in transfected groups of cells. Red: cleaved caspase-3, Blue: DAPI, Green: GFP-A53T α-syn. Scale bar: 2 mm. (F) Quantitative analysis of expression of A53T α-syn and cleaved-caspase 3, showing significant difference in the number of GFP and activated-caspase 3 positive cells compared with the single (A53T) transfection group (*n* = 3, *P < 0.05, **P < 0.01, ***P < 0.001, means ± SD). Around 100 cells were counted for each transfected group. (**G**) Western blot representing change in expression levels of inducible Hsp70, GFP-α-syn, and Bax as a result of overexpression of WT HSF1 and S303A/K80R/ K80Q mutant forms of HSF1, as well as RSV 20 µM treatment in A53T α-syn-transfected cells.

In support of the previously identified neuroprotective role of HSF1, we confirmed that overexpression of HSF1 (or HSF1 S303A, a constitutively activated form of HSF1) greatly reduced α-syn toxicity as measured by α-syn aggregation and reactive oxygen species (ROS) levels in A53T-trasnfected cells (Fig. 6A and B). Furthermore, downregulation of NEDD4 (or C866S NEDD4) resulted in similarly outcomes (Fig. 6A–D). On the contrary, NEDD4 overexpression led to increased caspase-3 activation, despite decreased α-syn aggregation from NEDD4 overexpression in cells overexpressing A53T α-syn (Fig. 6A–D). This is consistent with the results from western blot suggesting that the pro-apoptotic Bax was rather increased although α-syn accumulation was reduced upon NEDD4 overexpression (Fig. 2E). Finally, RSV was proven to be protective as reported previously (Fig. 6A–D).

Next, we compared the functional consequence of overexpression of HSF1 K80Q and K80R mutants on α-syn toxicity in comparison with WT and constitutively activated form of HSF1. Induction of Hsp70 has been pointed out as a key feature of SIRT1-mediated protection against α-syn aggregation via HSF1 activation (19). We confirmed that exogenous α-syn was significantly reduced as Hsp70 was induced by overexpression of HSF1 S303A and K80R, as well as by RSV treatment (Fig. 6E–G). However, HSF1 K80Q was found to neither reduce GFP nor alleviate A53T triggered toxicity (Fig. 6E–G). Taken together, these results fully agreed with our findings on the NEDD4-HSF1 axis involved in α-syn clearance.

## Discussion

Here we have provided compelling and novel *in vitro* and *in vivo* evidence that proteotoxic stresses, as exemplified by α-synucleinopathy, induce rapid and sustained HSF1 protein degradation mediated by the E3 ligase NEDD4 through UPS. We show here that both WT and A53T mutant α-syn caused HSF1 protein degradation to varying degrees: while A53T α-syn-induced HSF1 lost in both cytosolic and nuclear compartments, WT α-syn overexpression appeared to only induce loss of nuclear HSF1. Whether nuclear HSF1 is more vulnerable to proteotoxic stress remains unclear.

The consequences of losing HSF1, a master regulator of stress response for cell survival, can be detrimental in many ways. For instance, impairment of HSF1-mediated stress response, reflected by failed induction of chaperone proteins to neuronal stresses can significantly impact the onset, development, and progression of stress-related neurodegenerative diseases. Additionally, given that HSF1 is an integrated part of proteosomal functions, even partial loss of HSF1, can further compromise proteasomal degradative machinery. This results in a build-up of misfolded substrate proteins that are labeled to be degraded by UPS (21,22). We recently discovered that HSF1 is the major transcription factor for activating not only *hsp* genes, but also a number of genes pertinent to synaptic functions, such as *bdnf* (23). Therefore, loss of HSF1 is expected to compromise synaptic plasticity and memory function. Given its various significant functions, loss of HSF1 may underlie a fundamental mechanism of neurodegeneration, featured by proteinopathies. It should be noted that HSF1 protein degradation appears to be a process selectively induced by proteotoxic stress, and not heat stress (F.-F. Liao, unpublished data). In fact, heat stress induces HSF1 activation, and much of our understanding of HSF1 regulation has derived from the studies under heat shock conditions on various species: similarly to what an Hsp90 inhibitor does, heat shock induces an initial releasing step of HSF1 sequestration from Hsp90, followed by a series of phosphorylation, trimerization and nuclear translocation (4–8). Multiple post-translational modifications of HSF1 have been studied in relation to its activity state, including acetylation, sumoylation and phosphorylation (3), and we can now add ubiquitination to that list.

Proteostasis has been most widely studied under heat shock conditions, and this HSR most notably concerns the HSF1 complex (21). Proteostasis under proteotoxic stress, while less extensively studied, is believed to share much in common as HSR. However, it remains unclear how misfolded proteins are targeted for proteolysis under these conditions. Recently, Rsp5 and its mammalian homolog NEDD4 were discovered as the E3 ligases responsible for the increased ubiquitylation induced by heat stress. These ligases mainly target cytosolic misfolded proteins upon heat shock for proteasome degradation (24,25). Interestingly, temperature-sensitive Rsp5 mutants revealed dysfunctional HSF1 transcriptional activity, as well as reduced protein expression of HSF1, conceivably due to some kind of post-translational modification(s) (26). In addition, physical interactions between Rsp5 and HSF1 were detected under this experimental condition. This work provided evidence of a modulatory role of Rsp5, as in its regulation of HSF1 during mRNA export from nucleus (27); however, it did not provide direct support for Rsp5 being the E3 ligase.

Herein, we provide compelling biochemical evidence for NEDD4 as the E3 ligase for HSF1 ubiquitination and the subsequence degradation via UPS. NEDD4 is the prototypic HECT-type E3 ligase for a large family that has been conserved from yeasts (Rsp5) to humans. NEDD4 was first identified by genetically screening developmentally down-regulated genes in the early embryonic, murine central nervous system (13,14). Human NEDD4 exists in at least eight isoforms, resulting from alternative splicing that recognizes a proline-rich motif (PPxY or PY motif) not present in the HSF1 protein.

Strangely enough, NEDD4 was also reported to be the E3 ligase of α-syn in Parkinsonism via the endosomal–lysosomal pathway (28). Defective Rsp5/NEDD4 pathways were linked to α-synucleinopathy, which was supported by genetic screening in yeast (27,29). We consistently found that overexpression of WT NEDD4, but not the DN mutant form, resulted in a significant reduction of both ROS and α-syn aggregation (Fig. 6A and B). The seemingly contradictory roles of NEDD4 in the degradation processes of both toxic α-syn and protective HSF1 raise possibility of involving different mechanisms. Although our current *in vitro* data suggest that HSF1 is primarily degraded via UPS, we cannot rule out possible involvement of other major lysosomal pathways, particularly the chaperone-mediated autophagy (CMA). In addition, different isoforms of NEDD4 may ubiquitinate α-syn and HSF1, subjecting them to different subcellular compartments that likely involve different co-chaperone networks. Nevertheless, our study seeks to identify NEDD4 as an important E3 ligase, much like the C-terminus of Hsc70-interacting protein and *Parkin* in neurodegeneration.

Protein acetylation/deacetylation has been recently linked to protein regulation, especially via complex interplay with other forms of post-translational modifications (30). K208 and K298 acetylation by the acetyltransferase EP300 has been reported to regulate HSF1 protein stability under heat stress during reorganization of nuclear proteosomal network (31). Although it may be coincidental, acetylation has recently been identified as a mechanism regulating tau turnover (32), implying that complex interplay between different post-translational modifications represents a common mechanism neurons uses to fine-tune major classes of molecular processes. Our study distinguishes a strong correlation between an increased acetylation of HSF1 and its degradation, which is contrary to the relationship identified between acetylation and the degradation of FTDP tau (33). Deacetylation of Lys 80 residue by SIRT1 activation has been shown to activate the transcriptional activity of HSF1 via assessing its promoter occupancy rate (20). We demonstrate that the same Lys 80 acetylation state directly correlates with the ubiquitination levels and the stability of HSF1. The underlying mechanism is unclear. Since we observed opposite levels of ubiquitination on the K80R but not K80Q mutants, ubiquitination of HSF1 unlikely occurs on the Lys80. However, it is plausible that somehow highly acetylated Lys80 (e.g. K80Q) alters protein conformation, making it more accessible for NEDD4-mediated ubiquitination.

In summary, we discovered complete loss of HSF1 protein under various conditions of proteinopathies, suggesting that aberrant HSF1 protein degradation may represent a common and important key molecular mechanism underlying neurodegeneration. While HSF1 is increasingly being recognized as an important therapeutic target (3), it should be approached with caution, and HSF1's protein degradation should be taken into consideration. Pharmacological development of a feasible HSF1-stabiliing agent is thus of high importance. It can perhaps be used in combination with an HSF1-activating agent to combat neurodegeneration. Our study has also demonstrated the potential significance of SIRT1-mediated deacetylation in HSF1 stability, adding to the growing body of evidence of sirtuins in the regulation of proteostasis (34). Fully delineating the underlying mechanism of SIRT1-mediated proteostasis will facilitate development of this promising strategy.

## Materials and Methods

### Cell culture, plasmid transfection and reagents

SH-SY5Y cells were cultured in Dulbecco's modified Eagles' medium (DMEM) supplemented with nutrient mixture F-12 and 10% fetal bovine serum (FBS). Human embryonic kidney (HEK) 293 cells were grown in DMEM plus 10% FBS. Cells were transfected with plasmids using Lipofectamine 2000 (Invitrogen) and harvested 2 days after transfection. RSV, MG132, cycloheximide, NAM, sirtinol were all from Sigma-Aldrich. Chloroquine diphosphate sulfate was from MP Biomedicals. Plasmid constructs used in transient transfection include EGFP-C1-WT α-syn, pEGFP-C1-A53T mutant α-syn and empty vector pEGFP-C1; pcDNA-SIRT1 WT and mutant SIRT1 H355A; pRC-CMV-NEDD4 WT and NEDD4 C866S; HSF1 WT; HA-Ub. HSF1 LPKY deletion mutant, HSF1 K80Q and K80R were cloned by using a method of site-directed mutagenesis (Agilent Technologies)

### *In vivo* ubiquitination assay

Transfected cells were lysed in buffer (2% SDS, 150 mm NaCl, 10 mm Tris–HCl, pH 8.0) with 2 mm sodium orthovanadate, 50 mm sodium fluoride and protease inhibitors. The resulting lysates were boiled for 10 min and sheared by sonication. For immunoprecipitation, the HSF1 containing protein complexes were pull-down by anti-HSF1 antibody, washed and eluted from beads via boiling and subjected to immunoblot using anti-ubiquitin antibody (Enzo life sciences). Western quantification was based on the intensity of interested signal using densitometry and ImageJ software program.

### Cycloheximide chase assay

In SH-SY5Y cells, 100 μM (Sigma) cycloheximide was added for the indicated times in the presence or absence of MG132 24∼48 h after transfection of empty or A53T vector with or without SIRT1 WT constructs. Cell lysates were then prepared for further analysis.

### Immunofluorescence analysis

SH-SY5Y cells and mouse brain tissues were fixed in 4% paraformaldehyde, human brain was fixed in 10% formalin, all followed by sectioning and then blocking and incubation of primary antibody overnight and then AlexaFluor-conjugated secondary antibody. Human snap-frozen inferior parietal (Brodmann Area 39) samples used for the study were provided by the Biobank at University of Kentucky Alzheimer's Disease Center (35). Neuropathologic diagnoses were as described previously and inferior parietal lobe tissue was used because when that is affected one can be confident of the ‘diffuse/neocortical’ subtype of DLB (36). Fluorescence image were captured by confocal and fluorescence microscopy (Olympus).

### AAV-α-syn virus and α-syn transgenic mice

Mice were anesthetized by intraperitoneal injection of 10% chloral hydrate 3.5 ml/kg, and were stereotaxically fixed and injected with AAV2 or AAV5-CBA-α-syn virus into the right substantia nigra of brains (5.25 × 10^13^ vg/ml, 1.5 μl at 0.5 μl/min speed, anterior fontanelle 4.4 mm, sagittal suture (right) 1.3 mm, 8.5 mm under the skull). Viruses were generously provided by The Michael J. Fox Foundation for Parkinson's disease Research. WT α-syn transgenic mice were generated by Dr Cai's laboratory using the same strategy as described previously (18). Samples of different brain regions from single or double transgenic mice were used for biochemical examination.

## Supplementary Material

Supplementary Material is available at *HMG* online.

## Funding

This work was supported by NIH grants R01 AG031893, R21 AG041934, R21 NS083908 to F.F.L, R37 AG18440 to E.M., R21 NS085830, P30 AG028383 to P.T.N., by Alzheimer Association grant IIRG-11-204040 to F.F.L. and by NIH intramural research program AG000929 to H.-B.C. Funding to pay the Open Access publication charges for this article was provided by NIH grant AG049772 to F.F.L.

## Supplementary Material

Supplementary Data
