# Supplementary material for: NEDD4-mediated HSF1 degradation underlies α-synucleinopathy
Source: Hum Mol Genet. 2015 Oct 26;25(2):211–22. doi: 10.1093/hmg/ddv445 (PMC4706110; doi:10.1093/hmg/ddv445)
Supplement: Supplementary Data [file supp_25_2_211__index.html]

NEDD4-mediated HSF1 degradation underlies α-synucleinopathy — NEDD4-mediated HSF1 degradation underlies α-synucleinopathy — NEDD4-mediated HSF1 degradation underlies α-synucleinopathy — Supplementary Data 

# NEDD4-mediated HSF1 degradation underlies α-synucleinopathy

## Supplementary Data

Supplementary Data

- Supplementary Data - Pdf file
