# Supplementary material for: NEDD4-mediated HSF1 degradation underlies α-synucleinopathy
Source: Hum Mol Genet. 2015 Oct 26;25(2):211–22. doi: 10.1093/hmg/ddv445 (PMC4706110; doi:10.1093/hmg/ddv445)
Supplement: Supplementary Data [file supp_ddv445_ddv445supp.pdf]

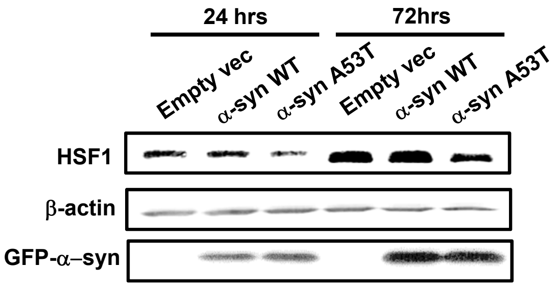

Fig. S1. HSF1 decrease in neuroblastoma cells overexpressing WT and A53T α-syn. Transfected cells were harvested at different time points, 24hrs and 72 hrs later . Protein lysates were subjected to Western blot analysis.

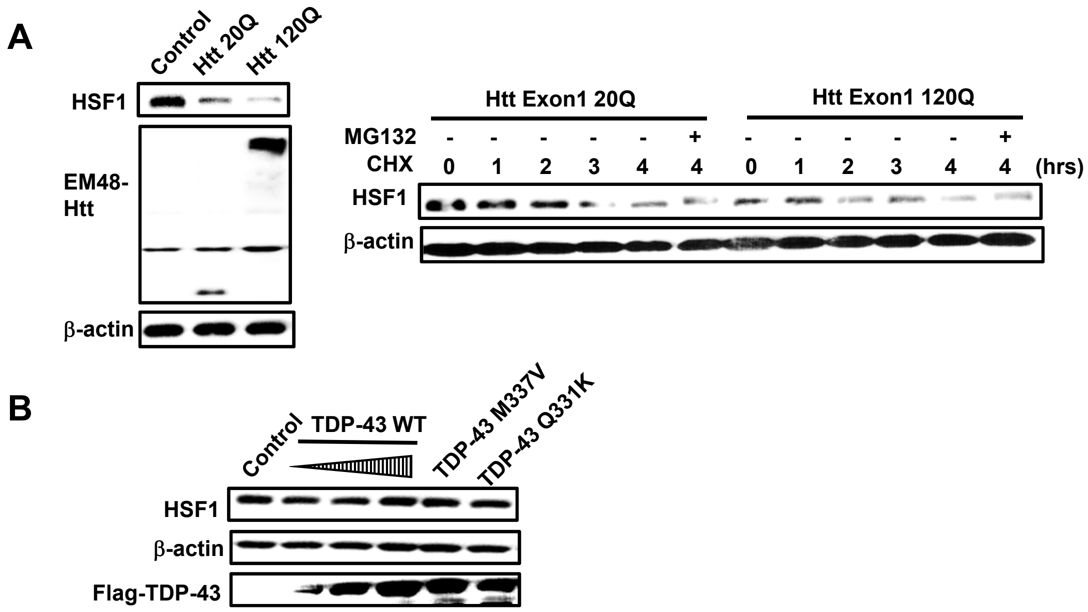

Fig. S2. Mutant Huntingtin (Htt) Exon 1 fragments, not TDP-43, decrease HSF1 expression levels. (A) Reduced HSF1 protein levels by Htt fragments. SH-SY5Y cells were transfected with Htt exon1 20Q or Htt exon 1 120Q. Untransfected cells were used as a control. Transfected cells were treated with 50 μM cyclohexamide (CHX) for various time points with or without pretreatment of 25 μM MG132. **(B)** No change on HSF1 protein levels by TDP-43. SH-SY5Y cells were transfected with increasing concentrations of TDP-43 WT, TDP-43 M337V, and TDP-43 Q331K.

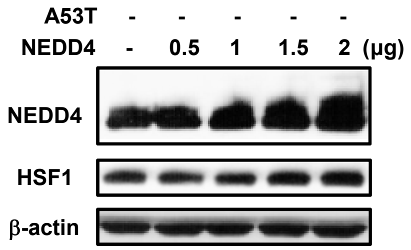

Fig. S3. No change on HSF1 by NEDD4 overexpression in cells not expressing A53T α-syn.

SH-SY5Y cells were transfected with different concentrations of WT NEDD4.

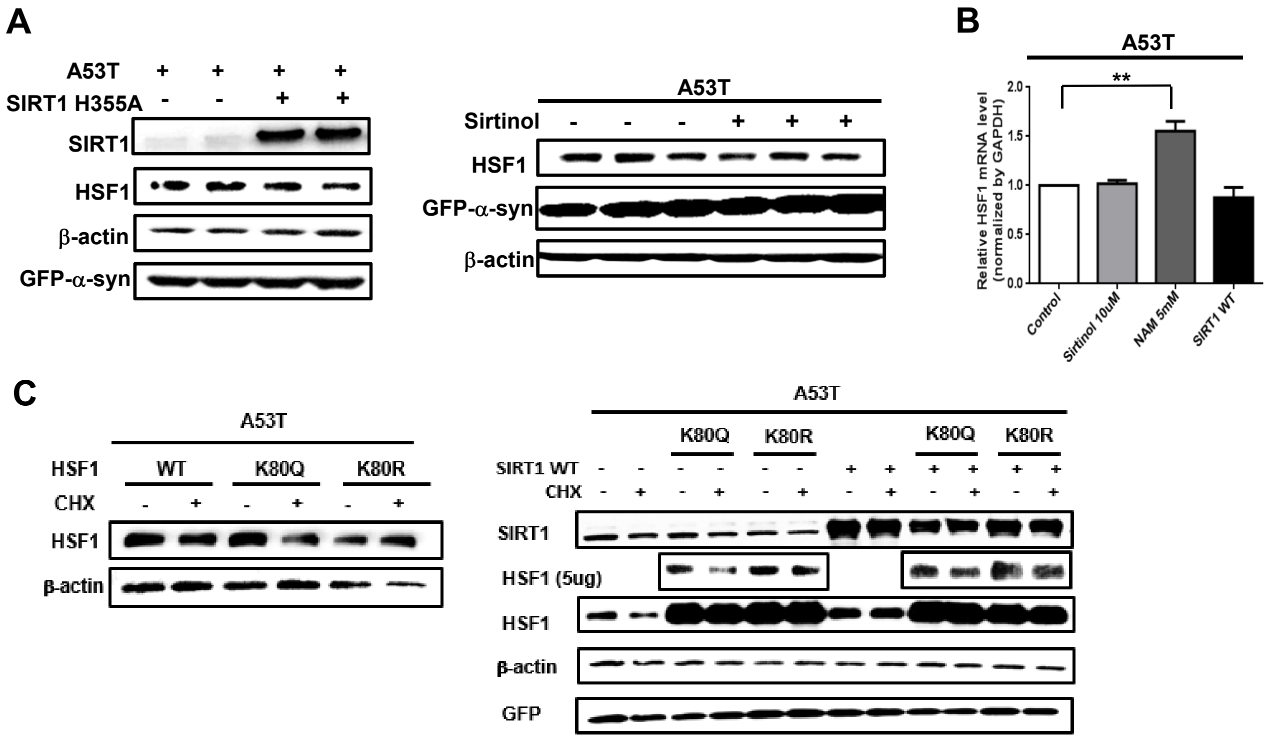

Fig. S4. (A) Effect of 10 μM sirtinol and overexpression of SIRT1 H355A on HSF1 levels. **(B)** Quantification of HSF1 mRNA levels were determined by q RT-PCR and normalized to GAPDH (n=3, **: P<0.01, means ± SD). **(C)** CHX assays. Cells overexpressing A53T were co-transfected with HSF1 WT or HSF1 K80Q or HSF1 K80R, followed by 50 μM CHX for 4 hrs at 24 hrs after transfection. Groups of A53T-transfected cells were co-transfected with SIRT1 WT and HSF1 K80Q/K80R and subjected to CHX assay (right).

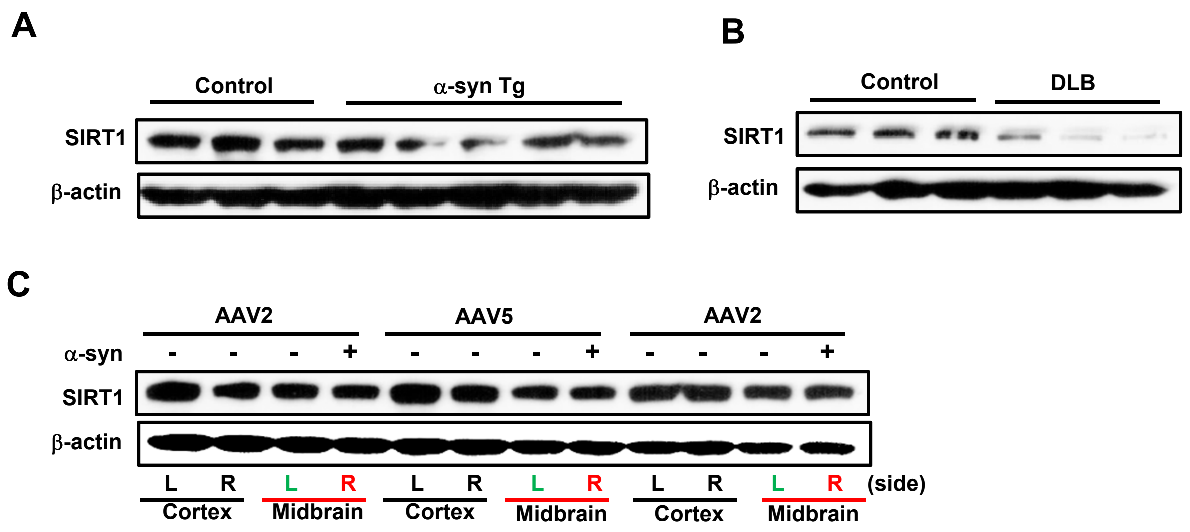

Fig. S5. SIRT1 protein expression in α-syn transgenic mice, human DLB patients and mice injected with AAV-α-syn viruses. (A) SIRT1 expression levels were generally decreased in α-syn transgenic mouse. **(B)** Reduced SIRT1 protein expression in the inferior parietal lobes of human patient specimens with diffuse Lewy body disease (DLB) compared to control without DLB. **(C)** SIRT1 expression was not altered by AAV-α-syn injection in the brain tissues of young adult C57BL/6 mice.
